# Challenges in the Development and Application of Organ-on-Chips for Intranasal Drug Delivery Studies

**DOI:** 10.3390/pharmaceutics15051557

**Published:** 2023-05-22

**Authors:** Muhammad Usman Khan, Xinyu Cai, Zhiwei Shen, Taye Mekonnen, Agisilaos Kourmatzis, Shaokoon Cheng, Hanieh Gholizadeh

**Affiliations:** 1 School of Engineering, Macquarie University, Sydney, NSW 2113, Australia; 2 School of Aerospace, Mechanical and Mechatronic Engineering, The University of Sydney, Sydney, NSW 2006, Australia; 3 Division of Pulmonary, Allergy and Critical Care Medicine, Department of Medicine, University of Pittsburgh, Pittsburgh, PA 15213, USA

**Keywords:** intranasal drug, organ-on-a-chip, in vitro drug tests, toxicology, nasal mucosa, nasal cavity, physiological relevance, in vitro tissue models

## Abstract

With the growing demand for the development of intranasal (IN) products, such as nasal vaccines, which has been especially highlighted during the COVID-19 pandemic, the lack of novel technologies to accurately test the safety and effectiveness of IN products in vitro so that they can be delivered promptly to the market is critically acknowledged. There have been attempts to manufacture anatomically relevant 3D replicas of the human nasal cavity for in vitro IN drug tests, and a couple of organ-on-chip (OoC) models, which mimic some key features of the nasal mucosa, have been proposed. However, these models are still in their infancy, and have not completely recapitulated the critical characteristics of the human nasal mucosa, including its biological interactions with other organs, to provide a reliable platform for preclinical IN drug tests. While the promising potential of OoCs for drug testing and development is being extensively investigated in recent research, the applicability of this technology for IN drug tests has barely been explored. This review aims to highlight the importance of using OoC models for in vitro IN drug tests and their potential applications in IN drug development by covering the background information on the wide usage of IN drugs and their common side effects where some classical examples of each area are pointed out. Specifically, this review focuses on the major challenges of developing advanced OoC technology and discusses the need to mimic the physiological and anatomical features of the nasal cavity and nasal mucosa, the performance of relevant drug safety assays, as well as the fabrication and operational aspects, with the ultimate goal to highlight the much-needed consensus, to converge the effort of the research community in this area of work.

## 1. Introduction

Intranasal (IN) drug delivery is gaining increasing interest as a promising alternative to intravenous and oral drug delivery. Even though IN drug delivery is mostly used for locally acting drugs, systemic therapies and the treatment of central nervous system (CNS) disorders via this route of drug administration have also gained significant interest [1,2]. IN drug delivery has many advantages over oral and intravenous routes of drug administration as it is non-invasive and provides a large, vascularised surface area, which promotes efficient drug absorption for systemic circulation. The above is associated with a rapid onset of action and, importantly, a higher drug bioavailability than the oral route as it avoids the hepatic first-pass metabolic effect [3]. A plethora of research has been conducted to optimise the efficiency of this drug administration method in terms of nasal device technologies, formulation development, and precise targeting of the drug’s action site inside the nasal cavity [4]. In the meantime, the need for developing physiologically relevant in vitro platforms for studying the therapeutic efficacy, toxicology, and delivery of IN drugs has attracted attention, and organ-on-chip (OoC) technology is considered a potential solution. However, designing and fabricating an OoC model closely relevant to the human nasal airway physiology remains a critical challenge.

OoC technology may be defined as the extended version of microfluidics that intend to mimic the multicellular architecture, chemical, and biomechanical microenvironment of human organs within a microscale 3D structure [5]. These platforms are microengineered to closely emulate the physiological functions as well as the pathology of human organs and can potentially be used for in vitro drug tests with an enhanced potency of emulating the observations in clinic [6]. At an advanced level, a network of multiple tissue types in the human body may be recapitulated by engineering the interconnection of multiple OoC platforms, mimicking different organ tissues [7].

The following section summarises the physiological environment of the nasal tissue at the microscale to provide insights for future work, such as recapitulating these anatomical structures in the design of microfluidic devices. Further, examples of various applications of IN drugs for local, systemic, and CNS therapies are reviewed to provide an overview of the potential applications of such OoC devices to examine IN drug delivery efficacy in vitro. The major side effects of IN drugs are classified and some clinical examples are summarised. This is deemed critical given that OoC devices have also found promising tools to simulate the clinical side effects at the early stages of in vitro drug tests. To the best of the authors’ knowledge, no OoC device has been developed for the human nasal airway, and a device that closely mimics the physiological characteristics of the nasal tissue and its dynamic microenvironment is currently absent. Hence, herein, in addition to discussing the core challenges in developing OoC devices, we present the criteria needed to produce a device with strong in vitro–in vivo correlation (IVIVC) that can be used for IN drug testing purposes.

### 1.1. The Physiology of Nasal Cavity

**Figure 1:**
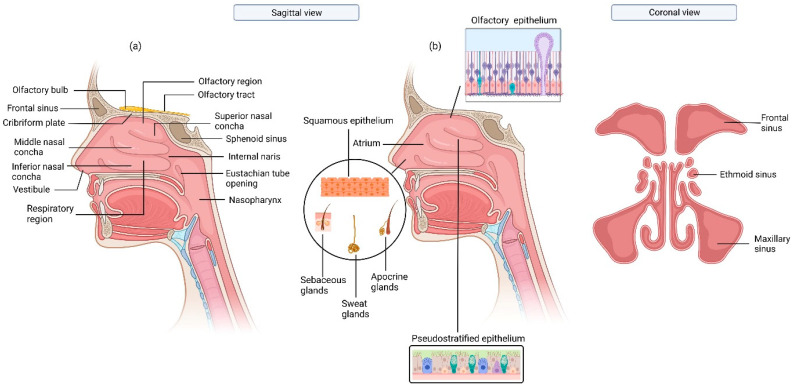
Anatomy of the nasal cavity, (**a**) the structure and different regions, (**b**) the various cellular composition of the epithelium, and the sinuses. Created with Biorender.com.

The nasal cavity is divided into three distinct regions: nasal vestibule (also known as the nostrils or external nasal valve), respiratory region, and olfactory region (Figure 1). The nasal vestibule is half covered with keratinized stratified squamous epithelium, which contains coarse hairs or vibrissae that filter inhaled particles larger than 5 µm [8] and is also half covered with respiratory epithelium, composed of pseudostratified ciliated columnar epithelial cells [9]. Just above the vestibules, the atrium is present, which is lined by squamous epithelium. Vibrissae (nose hairs), sebaceous glands, sweat glands, and apocrine glands are also present in this region [10]. The respiratory region constitutes the largest area of the nasal cavity, covered with ciliated pseudostratified epithelial and mucus-secreting goblet cells.

**Figure 2:**
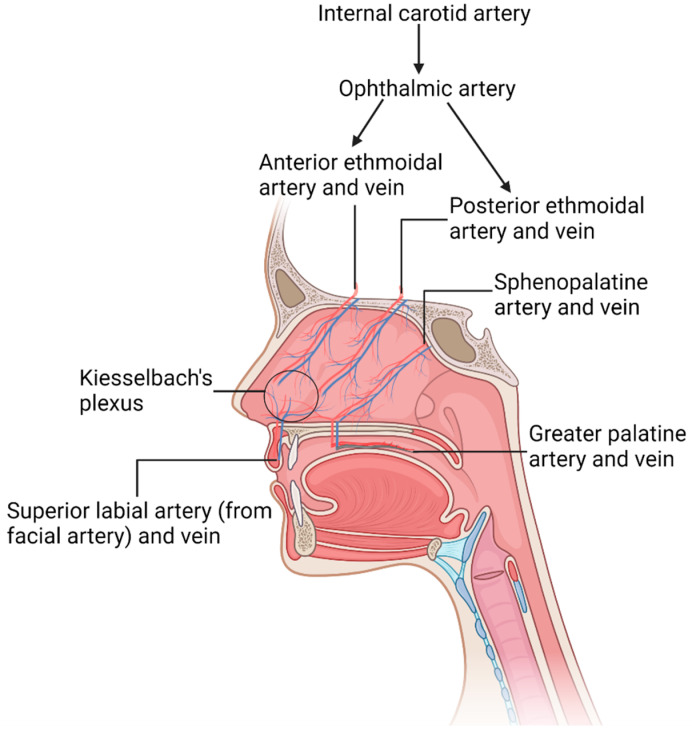
The illustration of the abundance of blood vessels supplying the nasal mucosa. Created with Biorender.com.

An abundant blood supply in the nasal cavity’s respiratory region warms and humidifies the inhaled air. This arterial blood supply originates from the internal and external carotid arteries [11], with further ramifications of the arteries breaking into a rich venous network in the nasal tissues. The thickness of the mat of veins in the nose varies between 1–5 mm depending on the region, with the greatest being in the conchae and the rhino pharynx regions. The internal diameter of veins in the nasal cavity ranges from 0.1–1 mm with many interconnections. The veins in the nasal septum have a diameter of 0.1–0.5 mm [12]. Figure 2 illustrates the blood vessels present in the nasal cavity. Ophthalmic artery is the primary internal carotid artery (ICA) branch that supplies blood to the nasal cavity. It divides into anterior ethmoidal arteries (AEA), posterior ethmoidal arteries (PEA), and dorsal nasal arteries (DN). AEA supplies blood to the nasal septum and lateral nasal wall. PEA supplies blood to the nasal septum and superior concha, while DN supplies blood to the external nose [13]. Another major artery that supplies blood to the external part of the nose is the facial artery (FA). FA stems from the external carotid artery and divides into the angular artery, bifurcating further to the lateral nasal artery and the superior labial artery [14,15].

**Figure 3:**
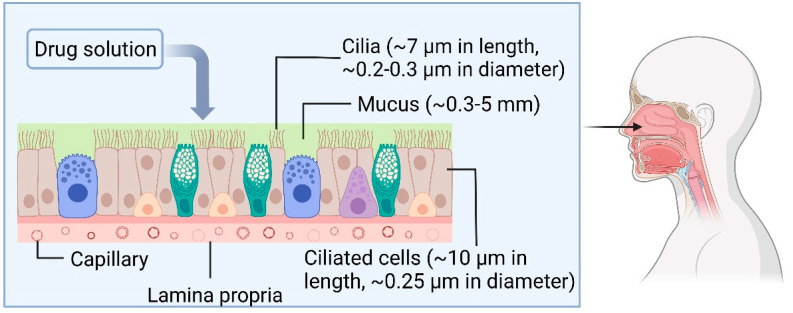
Illustration of the complex microenvironment of nasal mucosa, including the ciliated cells, mucus, and capillaries, which function as barriers against epithelial drug absorption. Created with Biorender.com.

The prevalence of veins in the nasal mucosa is favourable for effective systemic targeting. However, the nasal epithelium and mucus are a barrier for IN drugs to permeate to access the systemic circulation. The nasal mucus is 10–15 µm thick [10], and the nasal mucosa, consisting of epithelium, lamina propria, basement membrane, and capillaries, has a varying thickness between 0.3–5 mm at different regions of the nasal cavity [16]. For instance, the thickness of the mucosa in the human nasal septum adjacent to the nasal valve is ~5 mm, and decreases to ~0.5 mm at the inferior region of the septum adjacent to the inferior concha [17]. The hydrophilic viscous mucus layer limits drug diffusion to the epithelium and systemic circulation and filters large hydrophobic particles [18]. In addition, the cells in the respiratory region of the nasal cavity contain cilia that beat rhythmically, and mucus is propelled towards the pharynx to clear the nasal cavity and paranasal sinuses. Hence, the inhaled particles are filtered through the nasal cavity by mucociliary clearance. An illustration of the nasal mucosa microenvironment is presented in Figure 3.

The olfactory region is lined with neuroepithelium and constitutes a small area of ~2–2.5 cm^2^ in the nasal cavity [19]. This is less than 2% of the total area of the nasal cavity, which has a surface area of 150 cm^2^ [10]. While the respiratory epithelial cells, covering all respiratory tract (excluding the larynx and pharynx), have 200 to 300 cilia per cell, the olfactory epithelial cells have far fewer cilia per cell than the respiratory region, which is likely to enhance the residence time of drug particles deposited at this region. Drugs are known to be transported across the olfactory epithelium via the transcellular, paracellular, and intracellular axonal pathways [2,20]. 

The rheological characteristics of the mucus can significantly affect the ciliary motion and, consequently, the mucus transport rate [21,22]. The viscosity of the nasal mucus may change in response to environmental factors such as temperature and pH. The physiological pH of the human nasal mucosa is approximately 5.5–6.5 [23]. The maintenance of a hydrated mucus layer within a narrow pH range is necessary for the proper functioning of mucus [24]. For example, sulphur dioxide (SO_2_) in a polluted environment can alter mucus pH as it causes mucus acidification, increases viscosity, and decreases the mucus flow rate [25]. Air inhaled at a relatively higher temperature could also increase the mucus flow rate [26]. In addition to environmental factors, disease conditions such as allergic rhinitis [27] and acute rhinosinusitis [28] may also affect mucus viscosity. IN drugs that decrease the nasal mucus’s dynamic viscosity and elastic modulus include mucokinetic drugs, such as acetylcysteine, deoxynucleoside I, sodium bicarbonate, and Alevaire [29].

### 1.2. IN Drug Delivery

IN drugs for local therapies are commonly liquid formulations administered by nasal spray/aerosol pumps. Such delivery systems are convenient to use, can target a wide distribution area of the nasal mucosa, and can help to humidify the nasal mucosa. However, the chemical and microbial stability of these formulations tends to limit their long-term usage and storage. The aqueous environment of liquid formulations is not only favourable for microbial growth, but also enhances the susceptibility of drug molecules to chemical degradations such as hydrolysis, which is the most common drug degradation mechanism that occurs as a reaction between the drug and water molecules whether the pH of the solutions is acidic, basic, or neutral [30]. Due to the potential microbial growth in such water-based formulations, adding preservatives might be unavoidable to improve the product’s shelf life, and these preservatives have been known to cause irritation and allergic reactions in the nasal mucosa [31]. As a result of chemical instability, the liquid formulations may have shorter shelf life than solid particles. In addition, these formulations mainly require certain storage conditions such as temperature. For the case of hydrolysis, storing liquid formulations at a low temperature (lower than room temperature) can help with preventing or slowing down the degradation process [32]. 

Contrary to liquid IN formulations, dry powders for nasal delivery are more chemically and physically stable and may exhibit longer residence time [33,34]. However, these formulations may not have the same distribution efficacy upon delivery compared to liquid formulations. The intranasally delivered antihistamines and corticosteroids are the most common localised nasal therapies to treat upper airway disorders such as chronic rhinosinusitis, seasonal rhinitis, and nasal congestion related to allergic reactions or infections, and sino-nasal polyposis [35,36]. Antihistamines and corticosteroids have low systemic bioavailability when administered orally. However, delivering these drugs through the IN route may improve the drug bioavailability and prevent the side effects associated with oral administration, such as sedation or the impairment of the psychomotor function [37]. 

The efficacy of systemic delivery via the IN route has been reported for propranolol, nifedipine, and nitroglycerin, all of which are used for cardiovascular indications [38]. The systemic drug delivery via the IN route may also be used to treat CNS-related disorders such as headaches, pain management, migraines, hormone replacement therapies, and therapies for emergencies such as seizure [39,40,41]. In these cases, the IN delivery of the drug targets the CNS after reaching systemic circulation. The IN delivery of morphine offers rapid and effective relief from pain when given to chronic cancer patients without subjecting the patients to extensive hepatic first-pass side effects [42,43], typically experienced when the drugs are administered orally. Additionally, compared to intravenous delivery, the IN delivery of morphine as a polar, hydrophilic, and low molecular weight drug can result in a relatively higher bioavailability [38,44]. The bioavailability of the intranasally administered morphine could be enhanced by 80% when formulated with absorption-enhancing agents, such as chitosan [42]. The mucosal permeation of IN drugs to target systemic circulation might be limited by the epithelial barrier of the nasal mucosa, including tight junctions (TJs). The paracellular spaces between the adjacent epithelial cells are composed of an epithelial junctional complex [45], including TJ proteins such as *zonula occludens-1* (ZO-1), ZO-2, and ZO-3. The barrier function of the nasal mucosa, however, could be impaired in some disease conditions, such as in individuals with chronic rhinosinusitis (CRS) [27], nasal polyps [46,47,48], allergic rhinitis [49], nasopharyngeal carcinoma under chemoradiotherapy (CRT) [50], as well as smokers [51] due to the lower expression of TJ proteins, activation of epithelial ion transport channels, or DNA damage to the nasal epithelium. The proinflammatory cytokines associated with the pathophysiology of CRS, nasal polyps, and allergic rhinitis such as interferon gamma (IFN-ɣ), interleukin (IL)-4, and tumour necrosis factor alfa (TNF-α) are found to disrupt the expression of TJ proteins, namely claudin-1 occludin [27,46,49]. A similar adverse effect on the expression of TJ proteins is also reported for smoker individuals [51]. Consequently, this will adversely affect the barrier integrity of the nasal mucosa. Studies have also shown that the nasopharyngeal carcinoma patients treated with CRT could have nasal epithelium with sloughing morphology and wider intercellular spaces as the side effects of the therapy. Moreover, a decreased population of basal cells and an absence or divergence of the cilia instead of sequentially arranged ciliated cells that exist in a normal epithelium are other factors observed in nasal epithelia exposed to CRT in vitro that can eventually result in epithelial barrier integrity dysfunction [50]. The means with which these diseases affect the underlying tissue structures and the permeation or efficacies of IN drug delivery at the molecular level merits further investigations.

The abovementioned physiological barrier factors that influence nasal drug absorption and systemic bioavailability may vary for different drugs depending on their physicochemical characteristics, e.g., solubility, hydrophilicity, and molecular weight (MW). The physicochemical characteristics of the drug may impact the drug absorption rate through the nasal mucosa, with some studies indicating that small molecules tend to have higher absorption rates than larger molecules (MW > 1 kDa) [31]. On the other hand, lipophilic drugs, for example, tend to have higher absorption rates than hydrophilic drugs [31,52]. Similarly, The pH of the drug solution can also influence the rate and extent of absorption, as the nasal mucosa has a specific pH range at which it functions optimally [53].

Nose-to-brain delivery is another potential use of IN drugs to target the CNS efficiently, given that oral administration, the most common route for drug administration, is ineffective in delivering drugs to the CNS to treat neurological disorders effectively. A primary reason for this difficulty is the blood–brain barrier (BBB), a highly selective junction between the CNS and its periphery composed of tightly connected endothelial cells. While the BBB protects the CNS against pathogens, neurotoxic molecules, and other potentially harmful substances in systemic circulation, it can adversely affect brain targeting via the systemic route. The transport of drugs from the nasal cavity to the CNS is through the olfactory region. Although many of the conceptual therapies are still under development or further investigations, the research has indicated a promising potential of nose to brain drug delivery to effectively treat or manage the progression of Parkinson’s disease [54,55,56,57], Alzheimer’s disease [58,59,60], and schizophrenia [61,62], where mechanisms of action for a majority of these drugs lie with accessing the brain tissue via the olfactory area, bypassing the BBB.

Therapeutic agents could be delivered through the IN route to the CNS to treat brain tumours, with fewer side effects found in the peripheral organs [63,64,65]. It has been shown that the IN delivery of chemotherapeutic agents such as perillyl alcohol, methotrexate, and telomerase inhibitors are effective alternatives to conventional drug delivery systems [66,67,68,69]. The IN delivery of RNA therapeutics is also investigated for treating neurodegenerative diseases. The delivery of stem cells through the olfactory tract to the CNS is another emerging application of nose-to-brain drug delivery [70], where the interest in pre-clinical studies on investigating the potential of stem cells or mesenchymal stem cells delivery to treat brain tumours using IN route is rising [63,64,71,72]. 

Neurodegenerative diseases, such as Alzheimer’s disease, Parkinson’s disease, and Huntington’s disease, are associated with the progressive degeneration and death of neurons, glial cells, and the neural network nerve cells in the brain and spinal cord. In addition, for many of these diseases, inherited forms or gene mutations are diagnosed [73]. Thereby, the RNA-based therapies are advantageous for treating such disorders as they enable controlling the gene expressions and targeting the disease-associated genes [73]. On the other hand, the delivery of stem cells to the affected areas can also promote the regeneration of the nerve cells. However, there remains challenges with both RNA and stem cell-based therapies. For instance, the therapeutic efficacy of stem cell delivery may be limited due to the immunorejection of the cells or the lack of good source of the cells [74]. Therefore, new strategies are being sought to overcome these challenges, such as improving the delivery systems by incorporating nanoparticles, liposomes, or the encapsulation of stem cells within hydrogels to provide mechanical support during the delivery, as well as enhance the cells’ survival and integration within the host tissue [75]. However, delivering these biomaterials via the nose-to-brain route requires further evaluations such as the targeting efficacy and local toxicity effects. 

Despite the above advantages of nose-to-brain drug delivery, targeting the olfactory region can be challenging for drugs delivered via the IN pathway due to the complexity of the nasal cavity geometry, which tends to produce low velocity, as well as a flow recirculation below the olfactory region, resulting in the minimal deposition of drug particles on the olfactory mucosa [76,77,78].

### 1.3. The Potential Application of OoCs for Toxicological Studies on IN Drugs

Despite having numerous advantages and therapeutic applications, IN drugs can also have side effects, and studying these effects in vitro can be an important application of OoCs in IN drug testing. One reason for the side effects of IN drugs might be the excipients that are usually used in the IN drugs’ formulations. Excipients are mainly added to IN formulations as preservatives, viscosity modifiers, emulsifiers, and buffering agents. Absorption enhancers and mucoadhesive agents are commonly used excipients in IN drug formulations. Absorption enhancers usually deliver large molecules, such as peptides and proteins, to improve their bioavailability and permeability across the nasal mucosa [79]. The role of mucoadhesive agents in nasal formulations is to prolong the residence time of the drug particles on the nasal mucosa for efficient drug absorption through the nasal epithelium via increasing the exposure time of the drugs. However, the safety profiles of the excipients can potentially affect the ultimate safety of the final formulation [79,80,81] and hence, their applications in IN formulations require toxicological studies independent from the active ingredients in the formulation. The updated list of excipients approved by the United States Food and Drug Administration (FDA) for IN formulations and their corresponding concentrations is provided online by the FDA website [82], and this is a helpful resource for toxicological studies regarding IN formulations. 

In the following, the side effects of IN drugs are categorized into systemic, CNS, pulmonary, and local side effects to provide examples of IN drugs’ side effects that may potentially be studies of OoCs in future research.

#### 1.3.1. The Systemic and CNS Side Effects of IN Drugs

IN drugs absorbed in systemic circulation may result in side effects in multiple organs. One example of IN drugs with systemic side effects is oxymetazoline, a topical decongestant. It is a sympathomimetic drug which can result in vasoconstriction (narrowing of the blood vessels), fast and irregular heartbeats, headache, dizziness, drowsiness, high blood pressure, hypertension, tachycardia, nervousness, and trembling [83,84]. Nephrotoxic effect is also reported for IN drugs such as streptomycin sulphate [85] and voriconazole [86].

Although the main advantage of CNS targeting via the IN route reduces systemic exposure, and the mitigated risk of systemic adverse effects [87], systemic and CNS side effects can still be observed for nose-to-brain delivered drugs. The IN administration of benzodiazepines, such as diazepam and midazolam, used to treat seizures and epilepsy, for example, may cause sedation, amnesia, and respiratory depression [88]. Nanotechnology-based aerosol drug system is an emerging technology for nose-to-brain delivery to facilitate targeted and efficient delivery to the brain, which has also been demonstrated to treat CNS disorders, such as psychosis and glioma and, importantly, to reduce the chances of side effects [89,90,91]. The plethora of research in this area is summarised by numerous review papers, where the different nanoparticles, therapeutic applications, and challenges are mainly discussed [92,93].

#### 1.3.2. The Pulmonary Toxic Effects of IN Drugs

Nasal aerosol products are characterised by particle size distribution and aerosol characteristics based on impaction studies, and these are performed to evaluate the potential risk of the particles’ being transported to the lower airway [94]. Despite the work taken to reduce the chance of IN drug’s bioavailability in the lower airways before approval, some adverse pulmonary effects are still reported for commercial IN products. An example of such side effects is the respiratory depression caused by the IN delivery of midazolam to manage epileptic seizures [88]. Benzalkonium chloride, commonly used as a preservative in nasal drug solutions, has also been demonstrated to cause pulmonary irritation, albeit being undertaken through animal studies [95]. This was demonstrated by the increase in inflammatory markers such as lactate dehydrogenase and glutathione-S-transferases and the expression of inflammatory cytokines such as IL-6 [96]. Indeed, the expression of inflammatory markers could be replicated in future OoC devices to test for toxicity, and this subject will be discussed in detail in the later sections of this review. 

#### 1.3.3. The Local Side Effects of IN Drugs

The side effect of nasal formulations involving the impairment of the physiological or biological characteristics of the nasal mucosa is classified as a local side effect. These side effects may vary between patients depending on factors such as the individual’s physiological factors, pre-existing disease, and environmental conditions, such as temperature and humidity [4]. The potential interactions of nasal drugs with the nasal mucosal cells and other anatomical structures, such as the mucus, cilia, nasal microbiota, and the produced enzymes or chemokines by the epithelial cells [97], need to be examined when evaluating the local toxicity of nasal drug formulations. In the following, examples of drug-induced toxic effects on the physiological characteristics of the nasal mucosa are discussed.

The mucus layer that covers the nasal epithelium helps transport inhaled particles via mucociliary clearance towards the nasopharynx. IN corticosteroids, antihistamines, and some commonly used preservatives may influence ciliary movements. Budesonide IN sprays may induce a reversible effect on ciliary beat frequency at different concentrations, while fluticasone propionate, levocabastine hydrochloride, and azelastine hydrochloride may result in a concentration-dependent toxic effect on the cilia [98]. The olfactory neuroepithelium contains Cytochrome-P450 (CYPs) enzymes, which can catalyse the metabolism of inhaled drugs [99], and the impairment of the nasal mucosal enzymatic activity may be caused by drugs such as lidocaine [100] and chlormethiazole [101]. 

The nasal blood flow plays an important role in regulating the temperature and humidity of inhaled air. Vasomotor drugs and corticosteroids are known to influence nasal blood flow. Oxymetazoline is a vasoconstrictor used as a nasal decongestant and can decrease the blood flow within the nose [83,102]. In contrast, histamine, albuterol, isoproterenol, and fenoterol may increase the blood flow of the nose [37,44].

## 2. Recent Technologies for In Vitro Studies on IN Drugs

The in vitro studies on the therapeutic effect and delivery efficacy of IN drugs are mainly based on using an air–liquid interface (ALI) culture of nasal epithelial cells, where the nasal mucosa cell layer models are usually prepared in donor–acceptor cell culture plates, e.g., Transwell^®^ or Snapwell inserts. In these conventional models, the cells are cultured on a flat permeable membrane under static fluidic conditions. To test IN drugs, a uniform layer of the formulation is usually applied on the nasal cell layer. These models are irrelevant to the native nasal mucosa tissue regarding the complex cellular structure, geometry, and fluid dynamics (velocity, pressure, and surface tension profiles), where the drug particles could be heterogeneously deposited on different sections of the intricate nasal cavity surface. The static condition of these current models is opposite to the dynamic microenvironment nasal mucosa in vivo, where cells are exposed to respiratory airflow (epithelium) or blood flow (endothelium).

While the interest in the delivery of drugs and vaccines via the IN route is increasing, the current testing models for IN drugs with such low in vitro–in vivo correlation (IVIVC) may not be reliable to predict the efficacy or toxicity of IN drugs such that they can match the outcome of the preclinical tests. Hence, 3D modelling, complex manufacturing techniques, and microfluidics have been used to develop models for human nasal cavity and nasal mucosa with physiological relevance for testing IN drugs.

### 2.1. Physiologically Relevant 3D Models of Human Nasal Cavity 

The transparent nasal cavity model (Koken Co., LTD., Tokyo, Japan) is an anatomically relevant model of the human nasal cavity that facilitates the studies on the IN aerosols’ performance and qualitative evaluation of the regional drug deposition in the nasal cavity. Due to the optical accessibility of this model, IN drug deposition can be assessed via imaging techniques and image analysis [103,104]. Another anatomical model of the human nasal airway is the Alberta Idealised Nasal Inlet (Copely, UK) with separable sections, including the vestibule, conchae, olfactory region, and nasopharynx. Contrary to the transparent nasal cavity model, the detachable sections of the Alberta Idealised Nasal Inlet enable quantitative evaluations of the regional IN drugs’ deposition [105]. 

Although testing IN drugs using these models sheds insights into the deposition pattern, none of the current models integrates with meaningful biological interfaces. Hence, their throughputs can hardly be used to infer meaningful therapeutic actions of IN drugs in vitro, especially concerning drug interactions with the cells. In addition, these models can hardly represent the nasal geometry of the wider population (e.g., age, gender, race) given that this can vary significantly between humans and is further complicated by diseases, such as nasal polyps.

### 2.2. Microfluidic OoC Models of the Nasal Mucosa

The microfluidic OoC technology is a potential solution to overcome the shortcomings of conventional in vitro tissue models by mimicking the physiological, biological, chemical, and biomechanical features of the tissues in vivo. There have been attempts to use this technology to emulate the dynamic microenvironment of human nasal mucosa in vitro [106,107,108,109], where the donor–acceptor structure has been used for the ALI culture of nasal epithelial cells.

The physiologically resembled gland-like structure of the nasal mucosa morphology was replicated in the epithelial compartment of a microfluidic chip, where epithelial and endothelial cells were co-cultured at the opposite sides of an extracellular matrix (ECM) channel. The model enabled the evaluation of cell–cell and cell–ECM interactions. As a result of epithelium–endothelium co-culture, the gland-inducing factors secreted in the endothelial cell compartment promoted the generation of gland-like structures in the nasal epithelial compartment of the chip, where mucin protein (MUC5b) and gland development marker (Sox9) were indicated [107]. 

Further, OoC models of human nasal mucosa have been used to study the potential effects of fluid flow on drug permeation across the epithelial barrier model. This was achieved by mimicking the drug particle flow in the epithelial compartment of the chip, as well as the systemic flow in the acceptor compartment [106,109]. These chips resembled ex vivo human nasal epithelium as they include the modelling of TEER, barrier function, and mucus secretion. In addition, integrating electrochemical sensors in the structure of the chips enabled the in situ real-time quantification of the drug permeation to the systemic flow.

The irritant effect of inhaled gaseous toxins on the nasal epithelium’s ciliary beating frequency (CBF) was demonstrated by a microfluidic chip fabricated by a modified Transwell^®^ insert, cultured with differentiated human nasal epithelial stem/progenitor cells, integrated into a PDMS-bonded cover glass. The CBF’s dose-dependent effect of gaseous formaldehyde was monitored in the chip using a microscope equipped with a high-speed camera [108]. While only a few OoC studies have focused on the toxicology of inhaled toxins in the nasal airway, there have been more studies on the application of OoC technology on the toxic effect of inhaled drugs and toxins on the lower airway or the acute and chronic toxicity of inhaled drugs on the liver was demonstrated either by lung [110] or lung–liver [111] replicas, respectively. Such studies can pave the way for future research on the fabrication of nose–lung models, emulating intranasally administered drugs’ pulmonary side effects.

A multicompartment airway-on-chip platform was fabricated with the interconnected nasal passage, mid-bronchial airway region, and acinar region of the human respiratory system. The airflow rate at each compartment was established based on a preliminary computational fluid dynamics (CFD) analysis to mimic the physiological airflow rate in the system. Its application for modelling the viral infection transmitted through the respiratory system was demonstrated by using the SARS-CoV-2 virus [112]. Future work is required to present the PK–PD relevance of this model to assess its suitability for toxicology studies. 

### 2.3. Challenges with Studying in Drugs Toxicity by OoC Models

To enable clinically relevant toxicology studies using OoC models of the human nasal mucosa, a significant enhancement of these models are required to predict the side effects of IN drugs. The potential challenges of developing advanced models closely relevant to the native nasal tissue are elaborated as follows.

#### 2.3.1. Mimicking the Cellular Architecture and Tissue–Tissue Crosstalk

Mimicking the heterogeneous nasal epithelium is required to model the native nasal mucosa-on-a-chip. The cellular composition of the nasal epithelium varies from a stratified squamous epithelium to a pseudostratified columnar ciliated epithelium, depending on the location. A stratified squamous epithelium covers the vestibule, the inferior meatus (the area beneath the inferior concha), and the pharynx. However, a larger portion of the nasal mucosa, including the conchae and the nasal septum, is lined by a pseudostratified columnar ciliated epithelium, which includes ciliated and non-ciliated columnar cells, basal cells, goblet cells (mucus segregating cells), and brush cells. The apex of the nasal cavity, i.e., olfactory region, is covered by the pseudostratified columnar olfactory epithelium constructed of bipolar olfactory neurons, sustentacular cells, and basal cells. In addition, tubuloalveolar Bowman’s glands exist in the lamina propria of the olfactory epithelium [113,114].

In addition, the interactions between tissues (e.g., epithelium/lamina propria/capillaries or olfactory epithelium/olfactory bulb) and inter-organ crosstalk (e.g., nose–brain, nose–lungs, nose–kidney, and nose–liver) have yet to be simulated by the current nasal OoC models. Specifically, there is a need to develop such multi-OoC models that include nasal mucosa analogues such that these can be used to study the potential effects of IN drugs on the neighbouring tissues or the side effects observed in other organs in addition to the local effects and the interaction of the nasal mucosa with the drug treatments. Given that both the kidney and liver tissues are involved in detoxification processes, hepatotoxicity and nephrotoxicity are two major reasons for drug withdrawal from the market; the integration of liver and kidney analogues with the nasal mucosa by OoCs should hence be considered in future studies. To undertake this work meaningfully, it requires the implementation of accurate design parameters, e.g., surface area or volume of each compartment, and fluid mechanics, e.g., flow rate, so that the relevant in vivo pharmacokinetics–pharmacodynamics (PK–PD) [115], toxicokinetics–toxicodynamics (TK–TD), and the absorption–distribution–metabolism–excretion (ADME) of IN drugs can be mimicked. Data from clinical studies might be used to determine these parameters to design multi-OoCs [116]. One of the first body-on-a-chip devices was prepared by Shuler et al. The device consists of colon cancer cells, myeloblasts, and hepatoma cells, and the device was used to examine the cytotoxic effect of tegafur when metabolized by liver cells into 5-fluorouracil [117]. Another example worth noting is a system that involves cardiac, muscular, neuronal and liver modules, which was created to study the toxicity of acetaminophen, doxorubicin, valproic acid, atorvastatin calcium, and *N*-acetyl-m-aminophenol [118]. A platform of up to ten interconnected human organ replicas that involves liver/immune, lung, gut/immune, endometrium, brain, heart, pancreas, kidney, skin, and skeletal muscle was also presented in recent years, and its application to mimic the distribution kinetics (PK) of diclofenac in vitro was demonstrated [119]. Another potential advantage of using OoCs for drug safety studies is that tissue pathology and the relevant PK–PD of diseased tissues can be simulated. Improved knowledge in this area is critical to help predict drug response and toxicity in diseases.

In addition to developing OoCs, microfluidics technology has also been used to cultivate cells on two- and three-dimensional chip devices, described as cell culture on a chip. These chips have been used to develop microfluidic models of tumours and to study anti-cancer drug toxicity. For example, Chen et al. modelled potential metabolism pathways and the cytotoxicity of doxorubicin and paclitaxel in vitro using a microwell-based microfluidic chip [120]. The efficacy of doxorubicin was also tested by Fang et al. using a microfluidic device, where highly proliferative HepG2 cells were cultured in a 3D sidewall-attached droplet array [121].

In addition to the abovementioned challenges related to the fabrication and operation of multiple organs in one platform, the liver or kidney analogues to be included in such platforms require the organ-specific complex cellular composition and physiological functions, which are currently being studied in the liver and kidney-on-a-chip research studies. For instance, Jang et al. [122] included liver sinusoidal endothelial cells (LSECs), stellate cells, and Kupffer cells in the vascular channel of a liver-on-a-chip in addition to the hepatocytes in the parenchymal channel of a liver-on-a-chip. In addition to better mimicking the physiological liver function, such as albumin secretion, this model enabled the simulation of various drug-induced liver toxicity phenotypes, e.g., depletion of Kupffer cells, steatosis (retention of fat by hepatocytes and hypertrophy of stellate cells), cholestasis (hepatocellular accumulation of bile salts), and fibrosis caused by different drugs with varying toxicity mechanisms that target different cell types. Similar models are likely helpful in uncovering the unknown mechanisms of toxicity. Moreover, it will help with simulating the functions and toxicity of other tissues and organs while mimicking multiple organs, tissues, or cell types in one platform.

#### 2.3.2. Mimicking Complex Geometry of Nasal Cavity

**Figure 4:**
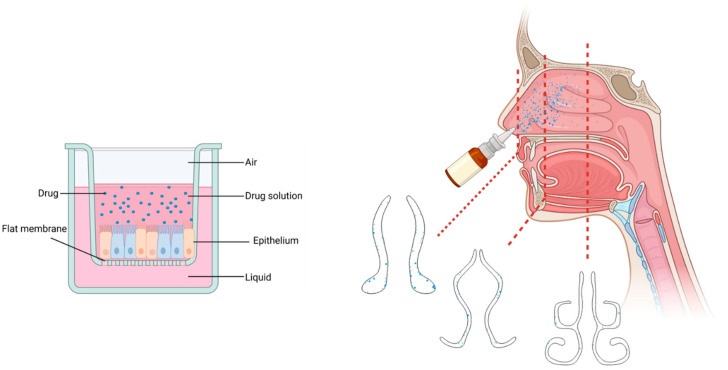
Illustration of the Transwell inserts as the current traditional nasal drug test platform with a flat membrane and even drug particle distribution along the cell layer (**left**) and the heterogenous deposition of nasal drug particles in the complex geometry of the human nasal cavity in vivo (**right**). Created with Biorender.com.

The current 2D in vitro nasal mucosa models, where the cells are cultured on a flat membrane, fail to represent the nasal airway’s intricate geometry and the 3D in vivo environment (Figure 4). Due to the use of such simple devices, complex airflow dynamics, such as the velocity and pressure profiles at different regions of the nasal cavity, and their consequential effects on the nasal spray and aerosol flow characteristics and sectional deposition patterns are ignored [123].

#### 2.3.3. Evaluation of IN Drugs’ Side Effects by OoCs

The analytical approaches to evaluate the drug-induced toxicity in OoC models may vary depending on the toxicity mechanisms or signalling pathway of the drug, where the biological characteristics and functions of cells and gene or protein levels are comprehensively assessed. Herein, some of the most common measurements reported in the literature to determine the drug-induced hepatotoxicity and nephrotoxicity by OoCs are summarised, which can be helpful for future toxicology studies in multi-OoC platforms involving nasal mucosa analogues.

##### Hepatotoxicity Assessments

The hepatotoxicity of drugs simulated by the OoC models involving liver analogue is evaluated by determining the cells’ survival (viability) [124,125] and tissue morphology [122] during inhibition in the expression or downregulation of the activity of metabolizing enzymes [126,127], i.e., CYPs enzyme family. This superfamily of enzymes, mainly found in liver cells [128,129], is involved in hepatic metabolism, catalysing a variety of biotransformations, metabolic reactions, and the bioactivation of drugs and pro-drugs. The liver dysfunction associated with liver injury is also evaluated by monitoring the decrease in albumin secretion [130], which is the essential function of hepatocytes that maintains the intravascular oncotic pressure. The increase in the release of liver injury biomarkers, such as miR-122 and keratin 18 [130], and reactive oxygen species (ROS) by hepatocytes, as well as the depletion of cellular glutathione (GSH) by both hepatocytes and non-parenchymal cells [122] are also observed in the liver-on-chip devices mimicking hepatotoxicity.

The drug-induced liver dysfunction is also evaluated by the elevation in the secretion of liver enzymes, including the alpha-glutathione-s-transferase (α-GST) and the transaminase family, i.e., alanine transaminase (ALT), aspartate transaminase (AST), alkaline phosphatase (ALP), and gamma-glutamyl transpeptidase (GGT) [122], the essential liver enzymes that catalyse the synthesis of amino acids. The liver inflammation could also be mimicked by the liver-on-chip, where the expressed inflammation cytokines, i.e., IL-6 and monocyte chemotactic protein-1 (MCP-1) by the primary hepatocytes, were quantified [122].

In addition to the abovementioned assessments and depending on the drug’s toxic mechanism, other analytical approaches may also be adopted. For instance, the inhibition of bile salts export pump (BSEP) in hepatocytes caused by bosetan was assessed by determining the intracellular accumulation of BSEP substrate, i.e., cholyl-lysyl-fluorescein (CLF) via fluorescent microscopic imaging and determining the decrease in BSEP protein and BSEP mRNA. In addition, the accumulated lipid in the hepatocytes observed in the steatosis phenotype of liver injury and the associated α-smooth muscle actin (α-SMA) expression within stellate cells was also monitored microscopically [122].

##### Nephrotoxicity Assessments

The drug-induced kidney injury has been assessed by OoCs in terms of apoptosis detection assays, e.g., live/dead cells staining, as well as the release of lactate dehydrogenase (LDH) [131,132]. The latter is performed as the increase in the urinary LDH efflux is associated with the acute kidney injury (AKI) [133]. The expression of genotoxicity markers, e.g., IL-6, CDK1, CCNA2, ATF3, MYC, and SRPX2 [126], is another method used to evaluate AKI on-chip. Furthermore, the damage to the filtration function of the glomerular endothelium as a barrier against large molecules is also evaluated on-chip, usually by measuring the permeation of fluorescein tracer IgG (MW = 150 kDa) and albumin (MW = 70 kDa) after drug treatment [134]. The disturbance of calcium homeostasis is another key factor known in the development of AKI, where the overload of the intracellular Ca^2+^ results in the tubular epithelial cells injury [135,136]. The increase in the intracellular Ca^2+^ release is modelled and evaluated by OoCs involving kidney replica exposed to ifosfamide, with known nephrotoxic effect when metabolised, by using Fluo-4 AM calcium indicator and obtaining fluorescent microscopic images of the cells that could be analysed for measuring the fluorescence intensity [137]. Importantly, the quantification of the injury-associated biomarkers such as kidney injury molecule-1 (KIM-1), osteoactivin, vascular endothelial growth factor (VEGF), and heme oxygenase 1 gene (HMOX1) by the OoC models is performed to evaluate nephrotoxicity by kidney tissue analogues on-chip. The induced oxidative stress in the cells in response to the drugs’ toxic effect is also evaluated by measuring the production of ROS by using fluorescent indicators of cellular and mitochondrial ROS, e.g., CellROX^TM^ and MitoSOX reagents [138]. Additionally, the urinary miRNA biomarkers observed in AKI patients has been quantified in the effluent of kidney analogue on-chips, which includes miRNA-21, -200c, -132, -155, -16, -24, and -30e [138].

#### 2.3.4. Fabrication and Operation of OoCs

Despite the improved throughput of drug tests by OoCs as discussed earlier, some challenges will remain with fabricating and operating these devices, including the expensive, time-consuming fabrication processes that sometimes fail to translate the clinical data. The unavailability of human organ-specific cells may also hinder the construction of the OoC platforms. While the marketed human-specific organ cells may not be stable for long-term use in culture media, potential ethical concerns may also be associated with using patient-derived primary cells. Human induced pluripotent stem cells (iPSCs) can be produced by using an individual’s genetic information to reprogram fibroblasts into stem cells. The iPSCs can be used as a promising alternative to evaluate disease mechanisms and the responses of organs to the therapies [139].

Another challenge in the fabrication of OoC models is related to the co-culture of different tissues in multi-OoC platforms. Maintaining different cell types in these platforms requires the perfusion of a universal culture medium [140]. To overcome this problem, a serum-free medium could be used. For example, Maschmeyer et al. used a serum-free medium in a study to investigate drug-induced toxicity in a four-interconnected OoC system that consists of the intestine, skin, liver, and kidney [141]. However, secreted biological factors may accumulate in the medium when it circulates for an extended period of time.

## 3. Conclusions

While the plethora of research on the application of OoC platforms for in vitro drug tests focuses primarily on simulating the physiology of human tissues such as liver, kidneys, and the lungs, only a few research studies have reported tests on IN drugs by such microengineered platforms. To the best of our knowledge, the in vitro drug tests by using such platforms have been limited to assessing the epithelial permeation of IN drugs and the areas such as toxicological studies. Therefore, in these studies, therapeutic efficacy evaluations have not been investigated. This is especially highlighted in this review given that IN drugs are potential to cause local, systemic, CNS, and pulmonary side effects. Some of these effects might even be observed after these IN products are marketed. Hence, the application of OoC technology for toxicological studies on IN drugs can significantly help with predicting such effects and lowering the risk of clinical side effects. Nevertheless, there remains challenges for the development of a relevant OoC platform for human nasal tissue, which has been discussed in this review in terms of mimicking the intricate geometry, complex cellular architecture, and the dynamic microenvironment of the nasal airway.

## Data Availability

Not applicable.
